# Michigan State Medical Society

**Published:** 1885-07

**Authors:** 


MICHIGAN STATE MEDICAL SOCIETY.

THE TWENTIETH ANNUAL MEETING OF THE MICHIGAN STATE MEDICAL SOCIETY occurred at Port Huron, beginning on Wednesday, June 10th, at 10:30 A. M.

The President, PROFESSOR DONALD MCLEAN, of Detroit, called the meeting to order, and prayer was offered by REV. HASTINGS BLISS, of Port Huron.

GENERAL HARTSUFF, of Port Huron, made a brief address of welcome.

Many prominent medical gentlemen from abroad were invited to seats upon the platform.

The Executive Committee made their annual report, which was discussed and approved.

The programme was so very full that those who had papers to read were requested to make them as short as possible, that all might be heard.

Dr. O. B. Campbell, of Ovid, read an interesting paper on "Migraine," which was discussed by Dr. Smart, of Hudson.

Dr. Woodworth, of Tecumseh, read a paper entitled "Treatment of Fracture." Dr. De Camp, of Grand Rapids, led in the discussion, which was interesting and spirited.

Dr. Ramsey reported that he had received a congratulatory telegram from the Maine Medical Society, which was then in session. The telegram was read, and a similar dispatch returned.

Dr. Hugh McCan, of Lapeer, read a paper on "Plaster of Paris in the Treatment of Fracture."

Dr. H. O. Walker, of Detroit, read a paper of great interest on "External Perineal Urethrotomy," which was discussed by Dr. Kimball, of Jackson.

The afternoon session was called to order by President McLean, and Professor McGraw, of Detroit, read an excellent paper "On the Origin of Cancers and Tumors." He presented a patient from whom he had removed the thyroid gland, and one patient from whom he had removed the entire left clavicle. These cases were of great interest and drew out quite a discussion.

Dr. Lundy, of Detroit, read a paper on "Iritis and its Relation to Certain General Diseases." Dr. Brodie opened the discussion, followed by Dr. Bennett and others.

At 4 o'clock, the discussion of the Treatment of Fractures was taken up by Dr. Kingston, of Montreal, followed by a number of gentlemen of large experience in this line of surgery.

Letters and telegrams of regret were received from Dr. Tye, of Chatham, Dr. Edwards, of London, Dr. Reeves, of London, Dr. Russell, of Toronto, Dr. Gribe, of Toronto, Dr. Dupuise, of Kingston, Dr. Beemer, of London, Dr. Woodruff, of London, Dr. Howard, of Montreal, Dr. Phelan, of Kingston, Dr. Covington, of Toronto, Dr. Burgess, of London, Dr. Rauch, of Springfield, Ill., Professor Goodale, of Philadelphia, and Dr. Jackson, of Chicago.

Dr. George K. Johnson, Chairman of the Committee on Reorganization, reported quite a number of important changes and recommended that they be adopted. The report was ordered printed and laid on the table for one year.

Dr. Casten, of Detroit, read a paper on "Sterility." It was discussed by Professor Palmer, of Ann Arbor, Professor Octerlooney, of Louisville, Ky., and others.

The evening session was called to order at 8:30 o'clock.

President McLean introduced Dr. Morse, of the Ohio State Medical Society.

Dr. Cook, of Muskegon, was called to the chair, and Professor McLean delivered his address, which was an able effort and well received.

Professor Palmer read a very exhaustive paper on "The

Physiological and Therapeutical Action of Alcohol." There was a large audience present to hear this paper, and the discussion of it was quite spirited. Dr. Whelan, of Hillsdale, led in the discussion, and afterwards read a paper entitled "The Possibility of a Concurrent Opinion upon the Physiological Effects of Alcohol."

The following gentlemen were proposed for membership: Dr. Merritt, of Fort Gratiot; Dr. Wilson, Port Huron; Dr. Chittock, Detroit; Dr. Smith, Detroit; Dr. Ingals, Detroit; Dr. Taylor, Loomis; Dr. Blanchard, St. Clair; Dr. McLaughlin, Cass City; Dr. Northrup, Port Huron; Dr. Stockwell, Port Huron; Dr. Clancey, Port Huron; Dr. Taylor, Mt. Clemens; Dr. Tibbitt, Linden; Dr. Chamberlin, Flint; Dr. Thompson, Flint; Dr. McIntee, Melin; Dr. Moors, Richmond; Dr. Ruch, Ypsilanti.

The Executive Committee at Port Huron consisted of Dr. Mills, Dr. Smith, Dr. McClintock, Dr. Plats and Dr. Stockwell, and these gentlemen had arranged to entertain all who were in attendance at the meeting.

Dr. Stockwell, of Port Huron, the first President of this Society, was warmly congratulated.

The Thursday morning session opened promptly at 9:30. The papers of Professor Palmer and Dr. Kinne, of Ypsilanti, were discussed at length and referred.

Dr. Smart reported a bill to Regulate the Practice of Medicine and Surgery, which was referred to a committee of five, and later in the day that committee reported favorably, and the Legislature will be asked to pass the bill.

Dr. Smart also suggested that a bill be prepared to the effect that all proprietary medicines must have their working formula on the labels and the bottles, and that the bill shall be prepared and presented to our next Legislature.

Dr. De Camp, of Grand Rapids, read a paper entitled "Germs in Medical Practice," and the paper was discussed by Dr. Shurley, of Detroit.

The following officers were elected for the coming year:

*President.*—Dr. E. P. Christian, of Wyandotte.

*Vice-Presidents.*—Dr. Patterson, of Charlotte; Dr. Griswold, of Grand Rapids; Dr. Carstins, of Detroit; Dr. Alvord, of Battle Creek.

*Secretary.*—Dr. Ranney, holds over.

*Treasurer.*—Dr. Smart, of Hudson.

The report of the Treasurer, Dr. Smart, is that the receipts of the year was $1,382.10; expenses, $407.33; on hand, $974.77.

Dr. Kinne, of Ypsilanti, read a paper on "Post Partum Hemorrhage."

Dr. Stockwell read a paper on "Functional Paralysis due to Malarial Poison."

Dr. Hemingway, of Kalamazoo, read a paper on "Students' Eyes."

Dr. George, of Ann Arbor, read a paper on "Abscess of the Cerebellum."

Dr. Sullivan, of Ann Arbor, read a paper entitled "Atresia of the Vagina with Loss of the Urethra and Base of the Bladder."

These papers were all discussed and referred to the Publication Committee.

At 4:30 the Society, and the invited guests, took the steamer "Conger" for a ride into Lake Huron, and returned to the Oakland House in the evening and enjoyed a fine supper, by invitation of Dr. Blanchard. Dr. McLean and Dr. Stockwell responded to the toast "Our ex-Presidents." Dr. Morse, Dr. Kingslin, of Montreal, and Dr. Octerlony, of Kentucky, responded to "Our Guests." Dr. Ward read a paper entitled

"The Professional Dude." "New Members," was responded to by Dr. Frothingham and Dr. White, of Detroit.

The Society returned to Port Huron at a late hour, each member delighted with Port Huron and satisfied that the present session had been one of the most satisfactory that the Society had ever held.

